# Identification of molecular signatures and pathways involved in Rett syndrome using a multi-omics approach

**DOI:** 10.1186/s40246-023-00532-1

**Published:** 2023-09-15

**Authors:** Ainhoa Pascual-Alonso, Clara Xiol, Dmitrii Smirnov, Robert Kopajtich, Holger Prokisch, Judith Armstrong

**Affiliations:** 1 Fundació Per La Recerca Sant Joan de Déu, Esplugues de Llobregat, Spain; 2 Institut de Recerca Sant Joan de Déu, Esplugues de Llobregat, Spain; 3 Institute of Human Genetics, Technical University of Munich, Munich, Germany; 4 Institute of Neurogenomics, Helmholtz Zentrum München, Munich, Germany; 5 CIBER-ER (Biomedical Network Research Center for Rare Diseases), Instituto de Salud Carlos III (ISCIII), Madrid, Spain; 6 Genomic Unit, Molecular and Genetic Medicine Section, Hospital Sant Joan de Déu, Barcelona, Spain

**Keywords:** Rett syndrome, MECP2 duplication syndrome, Rett-like phenotypes, Multi-omics, Transcriptomics, Proteomics, Differential expression

## Abstract

**Background:**

Rett syndrome (RTT) is a neurodevelopmental disorder mainly caused by mutations in the methyl-CpG-binding protein 2 gene (*MECP2*). MeCP2 is a multi-functional protein involved in many cellular processes, but the mechanisms by which its dysfunction causes disease are not fully understood. The duplication of the *MECP2* gene causes a distinct disorder called MECP2 duplication syndrome (MDS), highlighting the importance of tightly regulating its dosage for proper cellular function. Additionally, some patients with mutations in genes other than *MECP2* exhibit phenotypic similarities with RTT, indicating that these genes may also play a role in similar cellular functions. The purpose of this study was to characterise the molecular alterations in patients with RTT in order to identify potential biomarkers or therapeutic targets for this disorder.

**Methods:**

We used a combination of transcriptomics (RNAseq) and proteomics (TMT mass spectrometry) to characterise the expression patterns in fibroblast cell lines from 22 patients with RTT and detected mutation in *MECP2*, 15 patients with MDS, 12 patients with RTT-like phenotypes and 13 healthy controls. Transcriptomics and proteomics data were used to identify differentially expressed genes at both RNA and protein levels, which were further inspected via enrichment and upstream regulator analyses and compared to find shared features in patients with RTT.

**Results:**

We identified molecular alterations in cellular functions and pathways that may contribute to the disease phenotype in patients with RTT, such as deregulated cytoskeletal components, vesicular transport elements, ribosomal subunits and mRNA processing machinery. We also compared RTT expression profiles with those of MDS seeking changes in opposite directions that could lead to the identification of MeCP2 direct targets. Some of the deregulated transcripts and proteins were consistently affected in patients with RTT-like phenotypes, revealing potentially relevant molecular processes in patients with overlapping traits and different genetic aetiology.

**Conclusions:**

The integration of data in a multi-omics analysis has helped to interpret the molecular consequences of *MECP2* dysfunction, contributing to the characterisation of the molecular landscape in patients with RTT. The comparison with MDS provides knowledge of MeCP2 direct targets, whilst the correlation with RTT-like phenotypes highlights processes potentially contributing to the pathomechanism leading these disorders.

**Supplementary Information:**

The online version contains supplementary material available at 10.1186/s40246-023-00532-1.

## Background

Rett syndrome (RTT, OMIM#312750) is a severe neurodevelopmental disorder characterised by psychomotor regression after a period of normal early development. It mainly affects girls, who typically present with loss of purposeful hand use and expressive language, gait abnormalities and stereotypic hand movements. In addition, the main symptoms can be accompanied by a variety of other dysfunctions, such as seizures, breathing disturbances, scoliosis, impaired sleep patterns and abnormal muscle tone [1]. The diagnosis of RTT is mainly clinical and is based on a set of criteria that differentiate between typical RTT and three atypical variants with distinctive features: the preserved speech variant, the congenital variant and the early-onset seizure variant [2].

Most typical and atypical RTT cases are caused by loss-of-function mutations in the methyl-CpG-binding protein 2 (*MECP2*, OMIM*300005) gene, located on the X chromosome [3]. MeCP2 is a chromatin-associated protein that acts as a transcriptional regulator, both repressing and activating transcription, and is also involved in maintaining heterochromatin structure, regulating splicing through interaction with splicing factors and miRNA processing by binding to microprocessor components [1]. MeCP2 is expressed ubiquitously, but is especially abundant in mature neurons. MeCP2 has proved to be crucial for neuronal maturation, dendritic arborisation and synaptic plasticity [1].

Mutations in other genes have also been identified in patients with RTT. Pathogenic variants in cyclin-dependent kinase-like 5 (*CDKL5*, OMIM*300203) and forkhead box G1 (*FOXG1*, OMIM*164874) have been detected in a substantial number of cases with early-onset seizure and congenital RTT variants, respectively [4, 5]. Moreover, with the generalisation of next-generation sequencing (NGS), the number of genes associated with RTT has increased remarkably [6–8]. Some of these are novel findings whilst others have already been associated with different neurodevelopmental disorders or epileptic encephalopathies. Patients with overlapping phenotypes with RTT but who do not fulfil established clinical criteria are termed ‘RTT-like’. Therefore, any patient with a combination of RTT features can be described as RTT-like [6, 7].

MeCP2 levels are tightly regulated in the cells and not only a loss of function can have pathogenic effects. Chromosomal duplications at Xq28 encompassing the *MECP2* and *IRAK1* genes, leading to their gain of function, cause *MECP2* duplication syndrome (MDS), a neurological disorder characterised by intellectual disability (ID), infantile hypotonia, seizures, speech impairment and recurrent respiratory infections [9]. It mainly affects males, whilst penetrance in females is highly dependent on X-chromosome inactivation (XCI). Phenotypic variability is high in patients with MDS and potentially related to the size and content of the duplication, which is unique for each family [10]. However, a clear genotype–phenotype correlation has not yet been found.

One of the drawbacks in studying the downstream molecular effects of MeCP2 dysfunction is the lack of accessibility to samples of the primarily affected tissue, the brain. In the search for new tissues, skin fibroblasts have demonstrated greater consistency in gene expression and include more OMIM and neurologically relevant genes compared with whole blood [11, 12].

Around 70 experimental and repurposed drugs have been investigated for RTT, but there is no approved treatment yet [13]. In RTT clinical trials, the success of the tested drugs is evaluated by measuring the improvement in the symptomatology and quality of life of the patients. The lack of a biomarker complicates an objective quantification of the improvements derived from drug treatments. An efficient way to extract huge amounts of molecular data in order to find biomarkers could be by analysing the RNA profiles and proteome of the patients using multi-omics technology.

To date, no multi-omics analysis has been performed with RTT human samples and only one has been published with 4 RTT mice samples [14]. Here, we aim to fill that knowledge gap by studying a cohort of 22 patients with RTT, 12 patients with RTT-like and 15 patients with MDS. Integration of transcriptomics and proteomics data could be a promising approach to find new potential therapeutic targets and biomarkers.

## Material and methods

### Clinical and molecular characterisation

The study was approved by the Hospital Sant Joan de Déu (HSJD) ethical committee, Comitè d’Ètica d’Investigació Clínica-Fundació Sant Joan de Déu (CEIC; internal code: PIC-219-20). Sixty-two individuals (49 patients and 13 healthy age-matched controls) participated in this study and provided written informed consent. Patients were recruited after clinical and genetic confirmation of their pathology as described elsewhere [15]. Eleven out of the fifteen MDS patients were described in Pascual-Alonso et al. [16], and the four new patients were characterised in the same way. We studied 22 patients with RTT and mutations in *MECP2* (21 females and 1 male); 15 male patients with MDS; 12 patients with RTT-like phenotypes and mutations in *CDKL5* (1 female and 3 males), *FOXG1* (1 female and 1 male), *NR2F1* (1 female), *GRIN2B* (1 female), *AHDC1* (1 female) and 3 female patients without molecular diagnosis; and 13 healthy controls (7 females and 6 males) (Table 1). Clinical severity of patients with RTT and RTT-like phenotypes was measured using the clinical severity score designed by Dr Pineda [17].

**Table 1:** Composition of the studied cohort, which consists of individuals with Rett syndrome (RTT) with mutations in *MECP2*, *MECP2* duplication syndrome (MDS) and Rett-like (RTT-like) with mutations in different genes that are not *MECP2* and healthy controls. ‘Age’ and ‘Duration of disease’ are given in years; Mean ± Standard Deviation with available data (Additional file 5: Table S2)

Individuals	Age	Duration of disease	Female	Male	Total
RTT	9 ± 6	12 ± 2	21	1	**22**
MDS	7 ± 6	6 ± 6	–	15	**15**
RTT-like	12 ± 7	11 ± 7	8	4	**12**
*CDKL5*	9.5 ± 10	9 ± 10.5	1	3	**4**
*FOXG1*	9.5 ± 3.5	9 ± 3.5	1	1	**2**
*NR2F1*	9	8.7	1	–	**1**
*GRIN2B*	9	8.7	1	–	**1**
*AHDC1*	13	11	1	–	**1**
Unknown mutation	18 ± 5	17 ± 5	3	–	**3**
Healthy controls	18 ± 14	–	7	6	**13**

Skin biopsies from the 62 individuals were obtained, and primary fibroblast cell lines were established. Fibroblast lines were grown on plates with Dulbecco’s modified Eagle’s medium high glucose with glutamine, supplemented with 10% heat-inactivated foetal bovine serum and 1% penicillin, streptomycin and B amphotericin (all from Thermo Fisher, Waltham, MA, USA). Cultures were kept at 37ºC with 5% CO_2_ in a humidified atmosphere. When the cells reached 70–80% confluence, they were trypsinised and either re-sowed on new plates or harvested for subsequent RNA or protein extraction. Frozen vials from all the fibroblast lines were entrusted to the Biobanc ‘Hospital Infantil Sant Joan de Déu per a la Investigació’, which is integrated into the Spanish Biobank Network of ISCIII for the sample and data procurement.

DNA was extracted from fibroblast cell lines using the DNeasy Blood & Tissue Kit (Qiagen, Hilden, Germany) according to manufacturer’s instructions. XCI was performed in all female samples as described by Allen et al. [18]. XCI was considered skewed with an allele ratio of 80:20 or greater (Additional file 4: Table S1).

### RNA sequencing

RNA was extracted from cultured fibroblast pellets using the RNeasy Fibrous Tissue Mini Kit (Qiagen, Hilden, Germany) following the manufacturer's instructions. The obtained RNA was then measured with a NanoDrop spectrophotometer and examined in an agarose gel to check its purity and integrity.

To further confirm the quality of the isolated RNA and to diminish undesirable gene alterations due to cell stress conditions [19], we performed reverse transcription-quantitative polymerase chain reaction (RT-qPCR) of five genes that are part of the oxidative respiratory chain: *MT-CO1*, *MT-CO2*, *MT-CYB*, *MT-ND4* and *MT-ATP6*. First, 500 ng of total RNA was processed according to the manufacturer’s instructions, and double-stranded complementary DNA (cDNA) was generated in the presence of random hexamers using the SuperScript III First-Strand Synthesis SuperMix for qRT-PCR kit (Thermo Fisher, Waltham, MA, USA). Primers for the five mitochondrial genes and two additional housekeeping genes (*RPLP0* and *ALAS1*) were designed with Primer3 software [20] (Additional file 5: Table S2). The qRT-PCR was performed with PowerUp SYBR Green Master Mix (Thermo Fisher, Waltham, MA, USA) in an QuantStudio 6 Flex Real-Time PCR System (Applied Biosystems, Waltham, MA, USA). All reactions were conducted in triplicate and the average of each triplicate group was used for the analysis, which is based on the ΔΔCt relative quantification method. Three non-stressed control samples were added to the experiment to get the normalised values. Amplified product specificity was assessed via melting curve. All samples that overexpressed two or more genes more than 1.5-fold the values of non-stressed controls were discarded (Additional file 2: Fig. S2).

For each sample, 2500 ng of RNA was used for library preparation. Illumina’s TruSeq Stranded mRNA kit (Illumina, San Diego, CA, USA) was used following the manufacturer’s protocol. Libraries were quantified in a 4200 TapeStation (Agilent Technologies, Santa Clara, CA, USA) and their integrity was checked. Sequencing was performed on an Illumina NextSeq 500 sequencer and 75 base pair (bp) paired-end reads with around 40 million paired reads per sample successfully mapped to the reference genome. At least two healthy controls of the same sex as the patients were included in all the runs to enable normalisation and to control the batch effect.

To validate the RNAseq experiment, we chose 22 significantly differentially expressed genes (DEGs) and performed RT-qPCR as explained above with a total of 23 different samples (Additional file 2: Fig. S2).

### Differential expression analysis

RNAseq reads were aligned to the human reference genome (GRCh37/hg19) using STAR (v2.4.2a) in a strand-specific manner. Uniquely mapped reads were counted for each gene using the HTSeq package (v2.0.2) [21] with gene models from GENCODE release 29. A final count matrix for analysis was generated by averaging the values of raw counts from different replicates of the same sample. Counts per million mapped reads (CPM) were computed and only genes where more than 50% of samples had at least 1 CPM were kept.

We first inspected age, sex, biopsy origin and batch as possible covariates in the differential expression study by principal component analysis (PCA) and cluster analysis, but found no clear patterns in our samples (data not shown). PCA identified the primary sources of variation in our data. The first three principal components, explaining 18.8%, 16.1% and 7.4% of the variance, were subsequently used in the model construction for differential expression analysis with DESeq2 (v1.34.0) [22]. We used a Benjamini–Hochberg (BH)-corrected p-value of 0.05 to consider significant differences.

### Enrichment and upstream regulation analysis

Enrichment analysis was performed using the clusterProfiler (v4.2.2) [23] and ReactomePA (v1.38.0) [24] R packages. Both overrepresentation analysis (ORA) and gene set enrichment analysis (GSEA) were carried out, using only significant DEGs and all expressed genes, respectively. Potentially enriched terms were searched in Gene Ontology (GO) [25], the Kyoto Encyclopedia of Genes and Genomes (KEGG) pathway database [26], WikiPathways (WP) [27] and the Reactome pathway database (RP) [28]. All genes with CPM greater than 1 in at least 50% of samples and with an existing EntrezID were used as background (a total of 11,904 genes). The cut-off value for considering a significantly enriched term was 0.05 in BH-corrected p-value.

We considered upstream transcription factors (TFs) responsible for some of the differential expression changes observed in our data and used the ChIP-X Enrichment Analysis 3 (ChEA3) tool to identify them [29]. ChEA3 contains information about TF gene co-expression, association in ChIP-seq studies and co-occurrence in gene lists, which is used to predict upstream TFs involved in the regulation of the user inputted gene lists. The lists of DEGs resulting from differential expression analysis were fed to ChEA3 to predict the possible involvement of TFs in their dysregulation.

### Proteomics

Proteomics experiments were performed at the BayBioMS core facility at the Technical University of Munich (TUM) in Germany. Fibroblast cell pellets containing around 0.5 million cells were sent frozen. These cells were thawed and lysed with urea containing buffer and quantified using BCA Protein Assay Kit (Thermo Scientific, Waltham, MA, USA).

For the proteomics experiment, 15 μg of protein extract was reduced, alkylated and digested using Trypsin Gold (Promega, Madison, WI, USA). Digests were acidified, desalted and TMT-labelled following the protocol described by Zecha et al. [30] using the TMT 11-plex labelling reagent (Thermo Fisher, Waltham, MA, USA). TMT batches were organised to always include one reference sample that is common to all batches in order to enable normalisation. Liquid chromatography–mass spectrometry (LC–MS) measurements were run on a Fusion Lumos Tribrid mass spectrometer (Thermo Fisher, Waltham, MA, USA) operated in data-dependent acquisition mode and multi-notch MS3 mode. MaxQuant version 1.6.3.4 [31] was used for peptide identification, and protein groups were obtained. Missing values were imputed with the minimal value across the dataset [32].

### Differential expression in proteomics data

Prior to any analysis, MS data were adjusted with respect to one identical control sample that was present in each MS batch as described previously [32]. Recalibrated intensities were log-transformed for normalisation and proteins that were not detected in all samples were removed. An initial exploratory inspection of data by PCA and cluster analysis revealed that samples were grouped by MS batch (data not shown). Therefore, we carried out the differential expression analysis using the limma (v3.50.3) package [33] in R, including the MS batch as a covariate in the model to adjust for this confounding factor. We used the removeBatchEffect function from limma to recapitulate the exploratory analysis after batch correction and we observed no more clustering by MS batch. Finally, we took a nominal p-value of 0.05 as a threshold to define differentially expressed proteins (DEPs).

We carried out enrichment analysis just like we did for transcriptomics data. As a background, we considered the proteins that we detected in all samples with a valid EntrezID (a total of 5894 genes).

## Results

### Transcriptomic profiles in primary fibroblast cell cultures

First of all, we examined the similarity between the transcriptomic profiles obtained from primary fibroblast cell cultures and those from several brain areas, in order to understand how many of the molecular alterations that we identify could be extrapolated to neural tissues. We used publicly available data from the Genotype-Tissue Expression (GTEx) project and compared mean TPM (Transcripts per Kilobase Million) in fibroblast cultured cells and 11 brain areas: amygdala, anterior cingulate cortex, caudate basal ganglia, frontal cortex, cerebellar hemisphere, substantia nigra, hippocampus, hypothalamus, nucleus accumbens basal ganglia, putamen basal ganglia and spinal cord. 98.5% of detected transcripts (TPM > 0.5) in GTEx cultured fibroblasts RNAseq samples correspond to genes with some degree of expression in at least one neural tissue (Additional file 3: Fig. S3). More than 99% of the transcripts detected in our analysis are also reliably detected in GTEx cultured fibroblasts samples, indicating that the vast majority of the data that we are analysing may be extrapolated to biological processes occurring in the brain and therefore may impact neurological phenotypes.

### Characterisation of RTT-*MECP2* versus controls

**Fig. 1:**
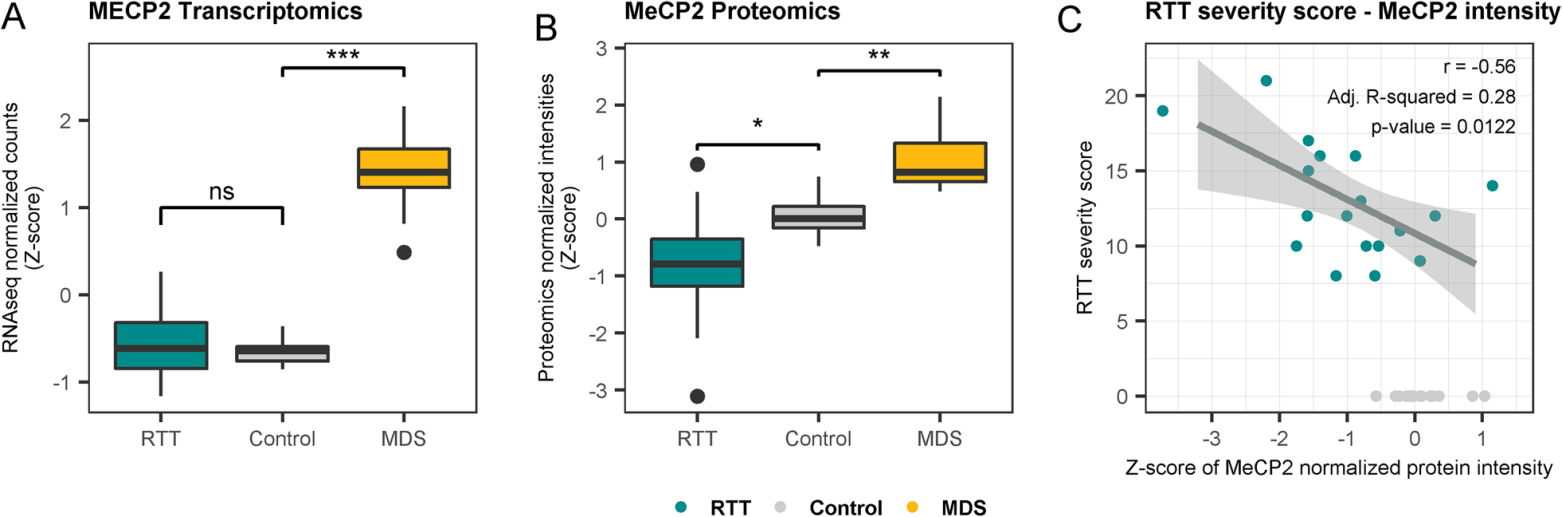
**A** MECP2 expression levels for RTT, MDS and control individuals obtained by RNAseq. **B** MeCP2 intensity levels for RTT, MDS and control individuals obtained by proteomics. **C** Pearson’s correlation between the severity score of RTT patients and MeCP2 levels

There were similar *MECP2* mRNA amounts in patients with RTT and controls, whereas MeCP2 protein amount was significantly reduced in patients with RTT (Fig. 1A, B). We found a significant correlation between MeCP2 levels and the Pineda clinical severity score of our patients with RTT, indicating that more severely affected patients present lower amounts of MeCP2 protein (Fig. 1C).

#### Transcriptomics and upstream regulator analysis

**Fig. 2:**
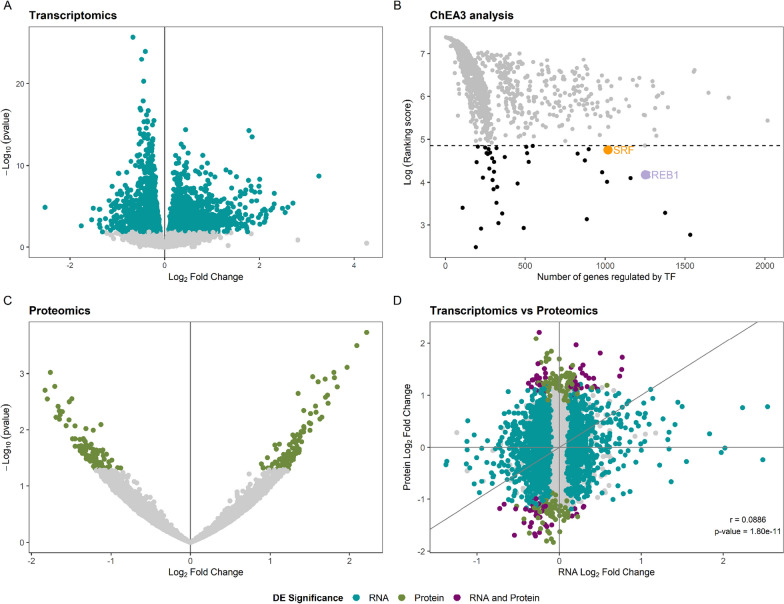
Summary of the results of the RTT-MECP2 versus healthy controls analysis. **A** RNAseq DE analysis results. The coloured genes are considered differentially expressed, passing a threshold of BH < 0.05. **B** Upstream TF ChEA3 analysis for the DEGs. The 40 TFs that were further studied are coloured in black. **C** Proteomics DE analysis results. The coloured proteins are considered differentially expressed, passing a threshold of nominal *p* value < 0.05. D) An integrated view of the transcriptomics and proteomics results. The genes that are significant at both analyses are coloured in purple

Differential expression analysis of patients with RTT carrying MECP2 mutations versus healthy controls showed 3446 DEGs: 1713 upregulated and 1733 downregulated (Fig. 2A). We subsequently used these DEGs as input for upstream regulator analysis with ChEA3. We inspected the top 40 ranked TFs searching for proteins that regulate a large number of the identified DEGs, since they would potentially be driving some of these transcriptomic alterations. The list of DEGs was significantly enriched in *CREB1* and *SRF* targets (Fisher’s exact test *p* < 0.05 in 5 of the 6 primary libraries in ChEA3). These two TFs have remarkable functions in neural tissues and could regulate the expression of 1253 and 1017 of the identified DEGs, respectively (Fig. 2B, Additional file 8: Table S5a). More than 98% of these potential targets have some degree of expression in at least one region of the nervous system, indicating that the alterations in transcriptomic networks identified in primary fibroblast cell cultures may affect the nervous system as well.

#### Multi-omics: integrating transcriptomics and proteomics data

Proteomics differential expression analysis revealed 224 DEPs, 123 upregulated and 101 upregulated (Fig. 2C, Additional file 6: Table S3a,b). Thirty-three and 28 of these are CREB1 and SRF targets, respectively. The number of DEPs is markedly lower than the number of DEGs identified in transcriptomics, probably in part due to the fact that mass spectrometry identified roughly half (5918) of the number of genes mapped in the RNAseq experiment (12,448). Almost 97% of the genes detected via mass spectrometry were also identified in RNAseq. Although the correlation between transcriptome and proteome differential expression findings was not high [Pearson correlation coefficient = 0.09, *p* = 1.8e-11, Fig. 2D], we found 75 genes deregulated at both the RNA and protein levels in patients with RTT (Additional file 6: Table S3C). The overlap between DEGs and DEPs is not significantly higher than expected by chance (Fisher’s exact test *p* = 0.1397, OR = 1.18), but some of the concordant genes constitute strong candidates for deciphering some of the pathomechanisms behind RTT, as well as for establishing biomarkers of this disorder (Table 2).

**Table 2:** Genes with concordant differential expression in transcriptomics and proteomics that are involved in the main biological processes identified via enrichment analysis

Gene	Direction	Biological process	Potential TF
*AFAP1*	Upregulated	Cytoskeletal processes	SRF
*FMNL2*	Upregulated	Cytoskeletal processes	CREB1
*FNBP1L*	Upregulated	Cytoskeletal processes	CREB1
*KIF3A*	Upregulated	Cytoskeletal processes	–
*MARCKSL1*	Upregulated	Cytoskeletal processes	–
*PLS3*	Upregulated	Cytoskeletal processes	SRF
*ARMC9*	Downregulated	Cytoskeletal processes	SRF
*ARHGEF1*	Downregulated	Cytoskeletal processes	–
*CDC42EP1*	Downregulated	Cytoskeletal processes	–
*IQGAP3*	Downregulated	Cytoskeletal processes	SRF
*PLXNB2*	Downregulated	Cytoskeletal processes	CREB1, SRF
*EIF4G3*	Upregulated	RNA processing	–
*NUDT12*	Upregulated	RNA processing	–
*SART1*	Downregulated	RNA processing	CREB1, SRF
*DDX31*	Downregulated	RNA processing	CREB1, SRF
*DDX54*	Downregulated	RNA processing	SRF
*MYBBP1A*	Downregulated	RNA processing	SRF
*NCALD*	Upregulated	Vesicular activity	–
*PREPL*	Upregulated	Vesicular activity	CREB1
*TMED1*	Downregulated	Vesicular activity	SRF
*ZFPL1*	Downregulated	Vesicular activity	CREB1, SRF
*AGPAT3*	Downregulated	Metabolism	–
*AACS*	Downregulated	Metabolism	CREB1
*CTBS*	Upregulated	Metabolism	–
*DCAKD*	Downregulated	Metabolism	CREB1
*HS2ST1*	Upregulated	Metabolism	–
*ORMDL2*	Downregulated	Metabolism	–
*PCK2*	Downregulated	Metabolism	–
*PI4KB*	Downregulated	Metabolism	–
*UAP1L1*	Downregulated	Metabolism	CREB1
*COMT*	Downregulated	Metabolism	CREB1

**Fig. 3:**
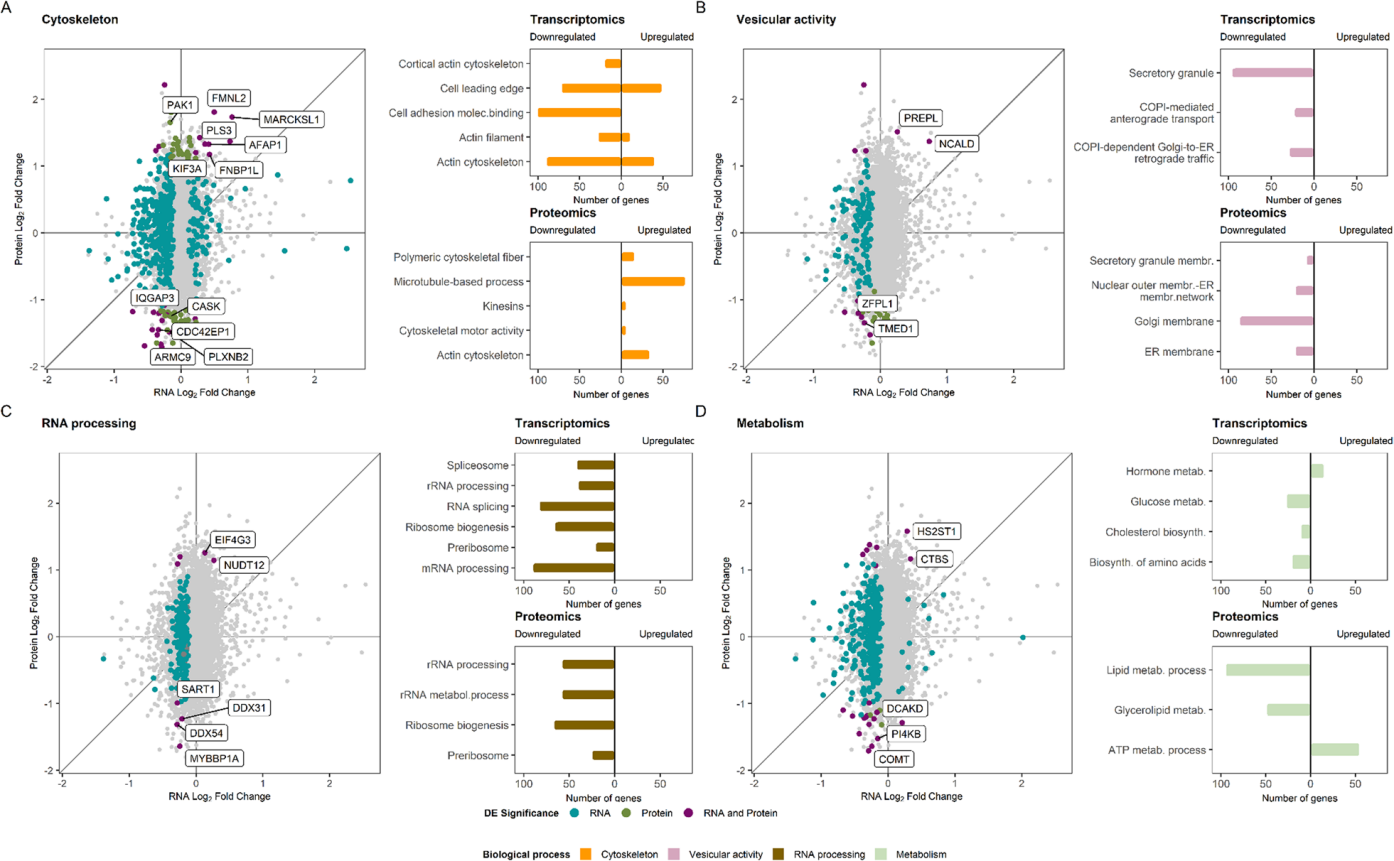
Summary of the enriched biological processes found in RTT-MECP2 versus healthy control analysis. **A** Significant DEGs and DEPs related to cytoskeleton (coloured in the dot plot) and the enriched terms found for those DEGs (upper bar plot) and DEPs (lower bar plot). **B** Significant DEGs and DEPs related to vesicular activity and the enriched terms found for them. **C** Significant DEGs and DEPs related to RNA processing and the enriched terms found for them. **D** Significant DEGs and DEPs related to metabolism and the enriched terms found for them

Enrichment analysis uncovered significant overrepresentation of genes and proteins involved in several cellular functions and processes, some of which may be extrapolated to neuronal tissues and thus are especially interesting when trying to elucidate the pathomechanisms underlying RTT (Additional file 7: Table S4a,b). The most remarkable pathways that repeatedly appeared significantly enriched with DEGs and DEPs were cytoskeletal processes, vesicular activity, rRNA processing and mRNA splicing (Fig. 3, Table 2). The vast majority of the consistent DEGs and DEPs driving this enrichment have some degree of expression in at least one brain area according to GTEx publicly available data.

### Patients with RTT versus patients with MDS

We compared the results of the DE analysis performed with patients with classical RTT and patients with MDS to identify common gene expression dysregulations that could shed some light into the pathomechanism underlying both syndromes.

**Fig. 4:**
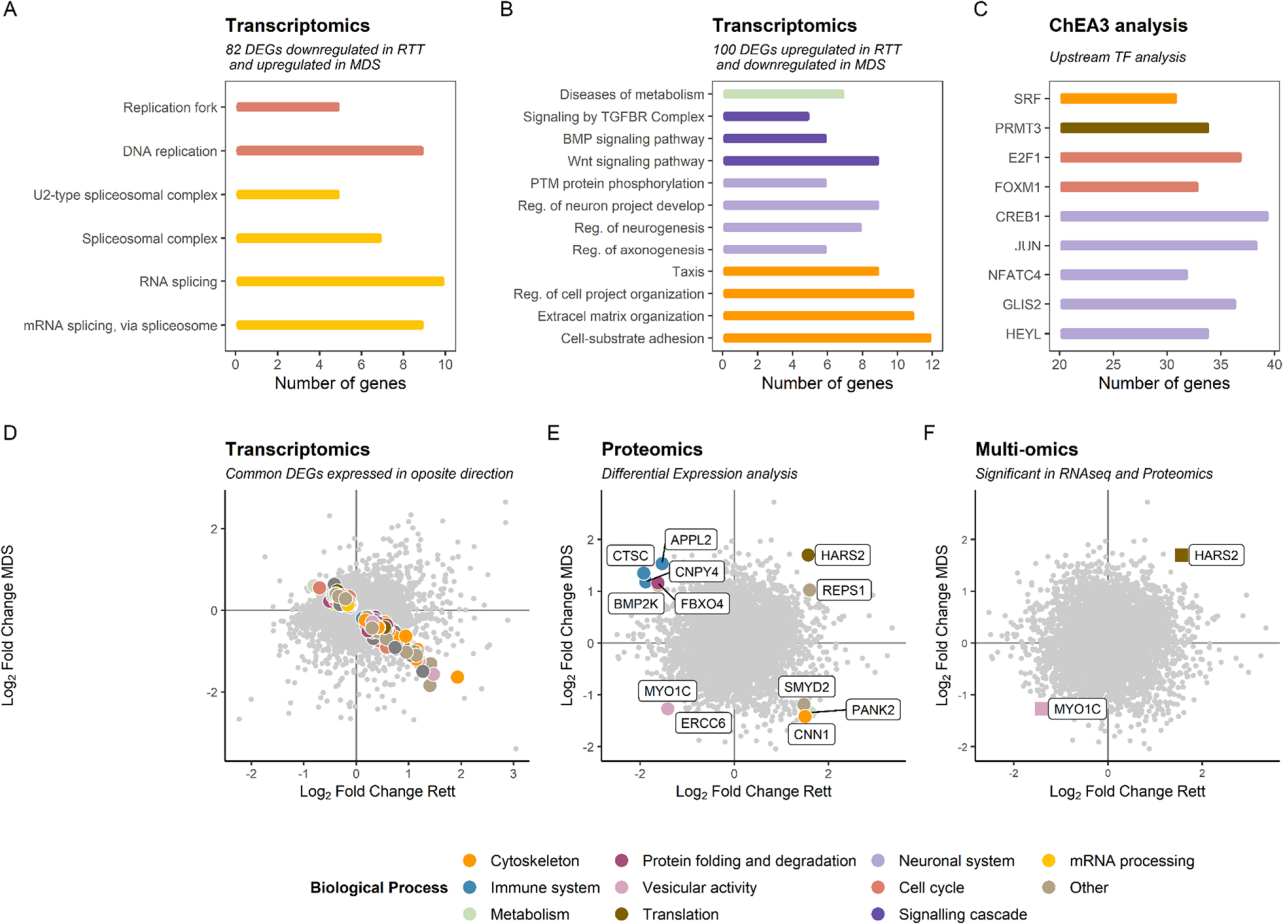
Summary of the common findings between the analysis of female RTT-MECP2 patients versus female controls and male MDS patients versus male controls. **A** Enrichment analysis results for the shared 82 DEGs downregulated in RTT and upregulated in MDS, coloured by Biological Process (BP). **B** Enrichment analysis results for the shared 100 DEGs upregulated in RTT and downregulated in MDS coloured by BP. **C** Relevant TF from ChEA3 analysis for the 82 and 100 DEGs dysregulated at transcriptomic level coloured by BP. **D** Transcriptomics DE analysis results. The common 182 DEGs expressed in opposite directions are coloured by BP. **E** Common 12 DEPs coloured by BP. **F** Common DEGs and DEPs obtained from a multi-omics approach

Transcriptomics DE analysis of male patients with MDS versus male controls and female patients with RTT versus female controls revealed 2465 and 3716 DEGs, respectively. Proteomics DE analysis returned 300 and 238 DEPs, respectively. Of these, 721 DEGs and 12 DEPs are shared between both groups, but only 2 genes are dysregulated with both omics in both syndromes (Fig. 4F). Those common DE genes are *MYO1C* and *HARS2*. *MYO1C* (OMIM*606538) is a myosin involved in cytoskeletal organisation and vesicle trafficking to the plasma membrane and is consistently downregulated. *HARS2* (OMIM*600783) is a mitochondrial histidyl-tRNA synthetase 2. At the RNA levels, it is consistently upregulated in patients with MDS and downregulated in patients with RTT. At the protein level, however, it is upregulated in both sets of patients.

Because *MECP2* expression is decreased in RTT and increased in MDS, we wondered whether they share DEGs that are expressed in opposite directions. In our cohort, 82 DEGs were positively correlated with MeCP2 expression levels (hence, upregulated in MDS and downregulated in RTT), and 100 DEGs were negatively correlated with MeCP2 expression (upregulated in RTT and downregulated in MDS) (Additional file 9: Table S6a,b). Enrichment analysis of those two gene sets revealed that pathways related to cytoskeleton and mRNA processing are altered. In addition, we found other molecular functions and pathways commonly altered between the RTT and MDS cohorts, some of which could help to understand why these two syndromes share clinical traits (Fig. 4A,B; Additional file 7: Table S4c).

The 82 DEGs downregulated in patients with RTT and upregulated in patients with MDS are overrepresented in terms related to mRNA processing and cell cycle (Fig. 4A, Additional file 7: Table S4c). mRNA splicing-related genes appear dysregulated in both analyses. Interestingly, 8 of the 82 DEGs are part of spliceosome complexes and another four are related to mRNA stability, processing and maturation functions. When looking at the ChEA3 TF enrichment analysis that regulates the same 82 DEGs, we found several TFs, most of them zinc finger proteins, described as cell cycle regulators and also *SRF*, which we found in the RTT ChEA3 analysis (Fig. 4C, Additional file 8: Table S5b). These results are consistent with our findings in transcriptomics enrichment.

The 100 DEGs upregulated in RTT and downregulated in MDS enrich processes related to neurogenesis regulation; signalling cascades, such as Wnt, BMP and TGFß; and the cytoskeleton (Fig. 4B, Additional file 7: Table S4c). TF analysis with ChEA3 for the 100 DEGs revealed that *CREB1* (BH < 0.05 in DE analysis) is upregulated and *SRF* is downregulated, and that they regulate 39% and 22% of the shared 100 DEGs, respectively. Moreover, the following TFs related to neuronal function are also enriched in the ChEA3 analysis: *HEYL, GLIS2, NFATC4* and *JUN* (Fig. 4C, Additional file 8: Table S5c).

Among the shared 12 DEPs, three, *APPL2*, *CNPY4* and *CTSC*, regulate immune response and are downregulated in RTT and upregulated in MDS (Fig. 4E, Additional file 9: Table S6c). Two other DEPs are related to cytoskeleton functions: *REPS1* and *CNN1*. *REPS1* (OMIM*614825) is a signalling adaptor protein that mediates cytoskeletal changes as endocytosis, and the protein is upregulated in both syndromes. *CNN1* (OMIM*600806) can bind to the cytoskeleton and produce smooth muscle contractions and is upregulated in RTT and downregulated in MDS.

### Patients with RTT versus patients with RTT-like phenotypes

Our RTT-like cohort was recruited considering their resemblance to the RTT phenotype. It encompassed nine patients with mutations in five different genes plus three patients without an established molecular diagnosis. The greater heterogeneity of this group complicated the identification of DEGs, as well as the interpretation of the differential expression results. Therefore, we established a significance threshold of BH < 0.1 for transcriptomics to be able to call DEGs despite the data heterogeneity. We interpreted these results in comparison with those obtained in typical patients with RTT, searching for shared molecular alterations that could constitute common grounds in the pathogenesis of overlapping disorders of diverse genetic nature.

**Fig. 5:**
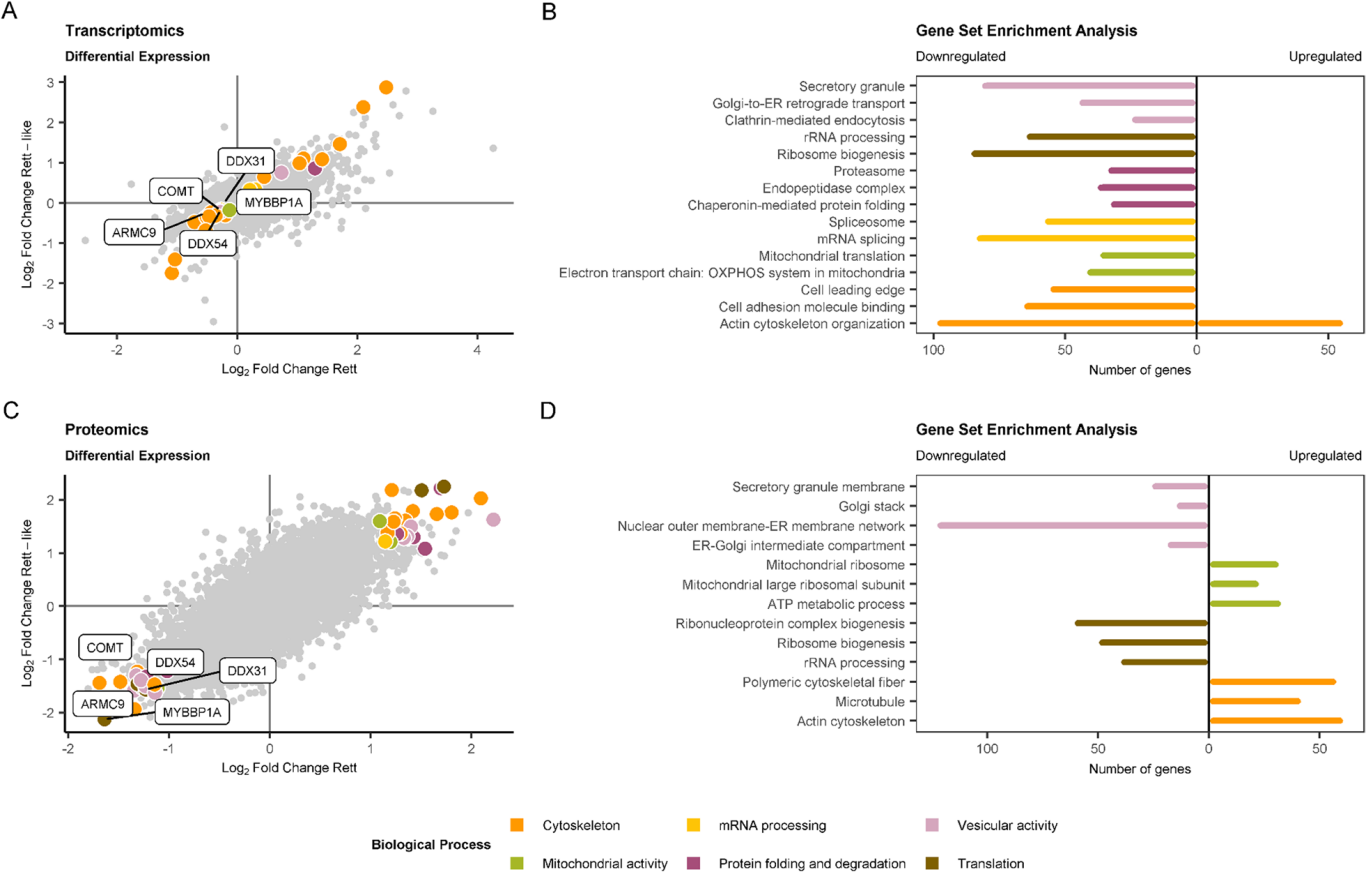
Summary of the common findings between the analysis of RTT-*MECP2* patients versus healthy controls and RTT-like patients versus healthy controls. **A** Common 63 DEGs (25 upregulated and 38 downregulated), coloured by Biological Process (BP). **B** Gene set enrichment analysis results for the shared 63 DEGs coloured by BP. **C** Common 81 DEPs (39 upregulated and 42 downregulated), coloured by BP. **D** Gene set enrichment analysis results for the shared 81 DEGs coloured by BP

DE analysis of transcriptomics data revealed 63 genes consistently altered in patients with RTT and RTT-like phenotypes (25 upregulated and 38 downregulated) (Fig. 5A, Additional file 9: Table S6d). SRF targets were significantly overrepresented in these common DEGs, with 31 putative targets out of 63 common DEGs. This could implicate SRF transcriptional regulation as a common mechanism linking the molecular phenotypes in RTT-spectrum disorders.

Proteomics data showed 81 proteins consistently dysregulated (39 upregulated and 42 downregulated) (Fig. 5C; Additional file 9: Table S6e), but no gene was altered in both transcriptomics and proteomics reaching significance. Nevertheless, some of the candidate genes identified in the multi-omics approach in RTT patients maintained a consistent dysregulation at the protein level in patients with RTT-like phenotypes [Table 3].

**Table 3:** Candidate genes identified in multi-omics analysis of patients with RTT and with a concordant alteration at the protein level in patients with RTT-like phenotypes

Gene	Direction	Biological process	Potential TF
*ARMC9*	Upregulated	Cytoskeletal processes	SRF
*DDX31*	Downregulated	RNA processing	CREB1, SRF
*DDX54*	Downregulated	RNA processing	SRF
*MYBBP1A*	Downregulated	RNA processing	SRF
*COMT*	Downregulated	Metabolism	CREB1

Transcriptomic and proteomic profiles of patients with RTT-like phenotypes are significantly correlated with those of patients with typical RTT (*r* = 0.69, adj-*R*^2^ = 0.47, *p* < 0.001 in transcriptomics; *r* = 0.75, adj-*R*^2^ = 0.56, *p* < 0.001 in proteomics) (Fig. 5A and C). Enrichment analysis of common DEGs and DEPs revealed terms related to cytoskeletal organisation, RNA processing, vesicular activity and metabolism, which constitute shared molecular alterations shared in patients with typical RTT and RTT-like phenotypes and could explain phenotypic overlap to some extent (Fig. 5B and D; Additional file 7: Table S4d-e).

## Discussion

### Multi-omics expression in RTT patients with *MECP2* mutations

**Cytoskeletal** actin filament-based processes play a crucial role in neuronal development, and their dysregulation is associated with cognitive disorders like RTT [34–37]. We discovered a significant enrichment of cytoskeleton-related DEGs and DEPs in RTT patients compared to healthy controls.

Our study found that *ARMC9* (OMIM*617612), a gene involved in cilium assembly, signalling and transport, was significantly downregulated in both mRNA and protein levels in patients with RTT. Its implication in cytoskeletal dynamics and the cytoskeletal abnormalities found in patients with RTT suggest a potential link between ARMC9 and RTT pathogenesis [38]. Scaffolding proteins, actin monomers and regulatory proteins were upregulated in RTT patients. We found an upregulation of p21-activated kinase 1 (*PAK1*, OMIM*602590), essential for regulation of the actin cytoskeleton and controls dendritic spine morphogenesis and excitatory synapse formation [39]. Moreover, Roux et al. [40] found an upregulation of proteins related to cytoskeletal motor activities, such as tubulin monomers and kinesins, that could be implicated in axonal transport to the neuronal growth cone. Our study also showed a downregulation of protein levels of Ca2 + /calmodulin-activated Ser-Thr kinase (*CASK*, OMIM*300172), a scaffolding protein that is involved in synaptic transmembrane protein anchoring in the brain [41]. CASK dysfunction is a promising route towards understanding some of the pathomechanisms behind RTT since it has been linked to neurodevelopmental disorders with overlapping phenotypes with RTT [42].

Another consistently downregulated mRNA and protein was *COMT* (OMIM + 116790), a methyltransferase required for the metabolism and degradation of catecholamine neurotransmitters, including epinephrine, norepinephrine and dopamine [43]. Patients with RTT and RTT mouse models have shown low levels of these biogenic amines, and alteration in dopaminergic metabolism has been associated with the characteristic motor deficits of RTT [44].

We also found a significant enrichment in genes and proteins related to **vesicular activity** located in the Golgi apparatus and the nuclear outer membrane–endoplasmic reticulum membrane network, as well as secretory vesicles. We found a significant upregulation of vesicular proteins located in neuronal axons. Prolyl endopeptidase like (*PREPL*, OMIM*609557) is a cytoplasmic protein with high expression in neuronal tissues. *PREPL* interacts with adaptor complex 1 (AP− 1), a protein complex that plays an essential role in vesicular trafficking [45]. NCALD is a neuronal calcium sensor protein that is involved in calcium signalling. It interacts with clathrin and actin and is involved in the modulation of endocytosis and synaptic vesicle recycling. NCALD was found to bind clathrin only at low calcium levels, resulting in inhibition and modulation of synaptic vesicle recycling [46]. Our study also found a significant downregulation of ZFPL1, a cis-Golgi membrane protein that regulates trafficking from the endoplasmic reticulum to the Golgi apparatus and maintains cis-Golgi structural and functional integrity [47].

We identified a downregulation of genes and proteins involved in **rRNA processing** and ribosome biogenesis in patients with RTT [48–50]. This could affect general protein translation in affected cells, possibly due to a reduction in mTORC1 activity [48, 49]. The downregulation of three proteins that interact with MeCP2, *MYBBP1A*, *DDX31* and *DDX54*, could explain alterations in rRNA processing and mRNA splicing [51]. The exact nature of the interaction between MeCP2 and these proteins is still unknown. We also observed downregulation of DEGs involved in **mRNA splicing** and spliceosomal complexes in patients with RTT. *MECP2* is known to interact with splicing factors [37, 52, 53], but a recent publication questions its role as a global regulator of splicing [54]. Additional studies are needed to clarify MECP2's role in splicing since many genes involved in mRNA splicing are repeatedly dysregulated in different transcriptomics experiments.

*CREB1* (OMIM*123810), which is a known MeCP2 interactor, regulates transcription in processes of relevance for neuronal survival and memory consolidation, among others [55, 56]. In astrocytes, it even regulates genes related to mitochondrial function, vesicle dynamics and the cytoskeleton [57]. Besides, one-third of our DEGs are regulated by *CREB1* and *CREB1* itself was significantly upregulated in our cohort at the mRNA level. *SRF* (OMIM*600589), which is an integrator of mitogen-activated protein kinase (MAPK) and Rho-GTPase-mediated signalling, regulates cytoskeletal dynamics. SRF binds to the serum response element (SRE) sequence, present in a subset of cytoskeletal genes such as *ACTB* and immediate early genes (IEGs) [58]. Besides, *SRF* regulates neuronal morphology and activity-dependent transcription [59] and suppression of *SRF*-mediated transcriptional responses has been found to produce a reduction in dendritic complexity in cortical neurons, which could contribute to the neuronal spine dysgenesis phenotype observed in patients with an RTT-spectrum phenotype [60].

None of the genes regulated in opposite directions in transcriptomics and proteomics were known MeCP2 partners. We analysed the functional relationships between them, but no clear biological processes were identified. We hypothesise that the discordance in transcriptomics and proteomics may be due to cellular compensatory processes.

### RTT and MDS: *MECP2* gene, two syndromes

Our study found that there are common genes between RTT and MDS. We found two shared genes, *MYO1C* and *HARS2*, which are a cytoskeletal component and a tRNA synthetase, respectively, in common significant DEGs and DEPs. The AKT/mTOR signalling pathway is downregulated in Mecp2 null models, indicating a deregulation of transcription followed by a limited ability to generate functional proteins [48]. Both syndromes seem to have a deregulation of the correct protein synthesis.

In total, 82 DEGs were downregulated in patients with RTT and upregulated in patients with MDS, indicating that *MECP2* is important for mRNA processing. Moran-Salvador et al. found a downregulated group of genes involved in DNA replication and cell proliferation in hepatic stellate cells of Mecp2-null mice and suggested inhibition of Mecp2 phosphorylation as a liver fibrosis treatment [61].

The 100 DEGs upregulated in RTT and downregulated in MDS revealed processes related to neurogenesis regulation, cytoskeleton, and Wnt, BMP and TGFß signalling cascades. The Wnt, BMP and TGFß signalling pathways are also involved in osteoblast activity and maintenance of cartilage [62–64]. Patients with RTT suffer from scoliosis, low bone mass density and a higher bone fracture rate than the general population [65, 66]. Scoliosis is the most commonly reported orthopaedic issue in patients with MDS, and osteopenia, contractures of joints and fractures have also been reported [67].

Our results detected four transcription factors related to neuronal function, *HEYL, GLIS2, NFATC4* and *JUN*. *HEYL* and *GLIS2* promote neuronal differentiation [68, 69], whilst *NFATC4* regulates adult hippocampal neurogenesis and shares a common signalling process with *BDNF* for neuron maturation [70, 71]. *BDNF* modulates many aspects of neuronal development, synaptic transmission and plasticity, and its dysregulation is found in RTT [72]. *JUN* plays a role in neuronal migration and axon–dendritic architecture, and its inhibition reduces breathing abnormalities in RTT mice and induced pluripotent stem cell neuronal models, and rescues the dendritic spine alterations [73].

Our findings indicate a resemblance of both syndromes at a molecular level, with several TFs involved in neural processes and dendritic complexity. Therapeutic strategies that seem promising for one syndrome could also benefit the other if the correct gene dosages are reached.

### TT-spectrum: one common clinical presentation, different mutated genes

The results of our study found that patients with RTT-spectrum disorders share common molecular alterations that could impact neuronal phenotypes. Almost one-third of the common DEGs are involved in cytoskeleton organisation and regulation, and some of these have important functions in neurons. The malfunctioning of cytoskeletal genes with prominent functions in neurite development could lead to neuronal spine dysgenesis and, consequently, to the emergence of disorders with common traits derived from this structural synaptic dysfunction [74, 75]. The enrichment in putative SRF targets among shared DEGs highlights the potential implication of SRF transcriptional regulation in RTT-spectrum common molecular alterations leading to overlapping phenotypes.

We also detected an overrepresentation of several terms related to nervous system development and structure, supporting that common molecular alterations found in patients with RTT-spectrum phenotypes can impact neuronal phenotypes. The downregulation of *ARMC9* observed in patients with typical RTT can also be observed in patients with RTT-like phenotypes, constituting a link between RTT-spectrum disorders and the overlapping phenotype caused by loss-of-function variants in this gene.

The patients with RTT-spectrum phenotypes in our study shared a downregulation of *SNRPC* expression at the RNA level that was not replicated in proteomics. This transcriptional alteration was also previously found in post-mortem brain tissue and embryonic stem cells of patients with RTT [49, 76]. *SNRPC* is a spliceosome component involved in 5’ splice-site recognition, so it may affect the splicing of many different targets and could constitute a shared mechanism of splicing dysregulation of patients with RTT-spectrum phenotypes. The dysregulation of splicing factors and regulators has been described in RTT as well as in other monogenic intellectual disabilities and in autism spectrum disorders (ASD) [77].

Protein translation may be affected in all patients with RTT-spectrum phenotypes. Several rRNA processing and ribosome biogenesis-related proteins found altered in patients with RTT were also consistently dysregulated in patients with RTT-like phenotypes, indicating this commonality. DDX54, DDX31 and MYBBP1A are MeCP2 partners and are linked to rRNA expression and preprocessing and could explain, at least to some extent, the shared dysregulation of ribosome biogenesis.

## Conclusions

Numerous studies have investigated the transcriptomes of individuals with RTT, resulting in over 60 published articles. Our study found that studying other human tissues, such as fibroblasts, can reflect the same dysregulations caused by loss of function of *MECP2.* However, integrating all knowledge is complicated by the heterogeneity in experiments and tissue-specific effects of *MECP2*. Dysregulation of various cellular functions was identified, including cytoskeletal organisation, vesicular activity, translation and mRNA processing, which are altered in patients with RTT, RTT-like phenotypes and MDS. *ARMC9* could be a potential biomarker for RTT and RTT-spectrum disorders. TF analysis supports *CREB1* and *SRF* as potential therapeutic targets. Shared dysregulated biological processes and cellular functions were found between patients with RTT, MDS and RTT-like phenotypes, with RTT and RTT-like being more similar than MDS. Further studies are necessary to validate these findings.

### Supplementary Information

**Additional file 1:** **Fig. S1.** Detection of fibroblast samples under oxidative stress conditions.

**Additional file 2: Fig. S2.** Comparison of the gene expression results between RNAseq and RT-qPCR.

**Additional file 3: Fig. S3.** Comparison of genes from Genotype-Tissue Expression (GTEx) project and mean TPM (Transcripts per Kilobase Million) in fibroblast cultured cells in RNAseq and RT-qPCR.

**Additional file 4: Table S1.** Detailed description of the study cohort.

**Additional file 5: Table S2.** Sequence of the designed primers.

**Additional file 6: Table S3.** RNAseq and proteomics differential expression results for RTT - MECP2 versus healthy controls.

**Additional file 7: Table S4.** Enrichment analysis results for the RTT-MECP2 versus healthy controls, RTT versus MDS, RTT versus RTT-like, transcriptomic and proteomic data.

**Additional file 8: Table S5.** ChEA3 upstream analysis results for RTT patients versus healthy controls, RTT versus MDS, RTT versus RTT-like.

**Additional file 9: Table S6.** Summary for the DEG and DEP from RTT-MECP2 vs MDS and RTT-MECP2 versus RTT-like.

## Data Availability

Our ethics approval and consent agreements allow us to share non-identifiable patient data and analysis data only, as such, we cannot provide BAM or VCF files. The analysis data provided are the gene expression count matrices, as well as the privacy-preserving count matrices of split and unsplit reads overlapping annotated splice sites from RNAseq. They will be available for download without restriction when this article will be published.

## Abbreviations

ASD: Autism spectrum disorder
BH: Benjamini–Hochberg-corrected p-value
bp: Base pair
*CDKL5*: Cyclin-dependent kinase-like 5
cDNA: Double-stranded complementary DNA
CEIC: Comitè d’Ètica d’Investigació Clínica-Fundació Sant Joan de Déu ChIP-X Enrichment Analysis 3
CPM: Counts per million mapped reads
DEG: Differential expression genes
DEP: Differential expression proteins
EEGs: Electroencephalograms
*FOXG1*: Forkhead box G1
GRCh37 (hg19): Homo sapiens (human) genome assembly GRCh37 (hg19) from Genome Reference Consortium
GSEA: GO: Gene Ontology
HSJD: Hospital Sant Joan de Déu
ID: Intellectual disability
IEGs: Immediate early genes
iPSCs: Induced pluripotent stem cells
KEGG: Kyoto Encyclopedia of Genes and Genomes
LC–MS: Liquid chromatography–mass spectrometry
MDS: MECP2-duplication syndrome
*MECP2*: Methyl-CpG-binding protein 2
NGS: Next-generation sequencing
NF-κB: Nuclear factor kappa B
ORA: Overrepresentation analysis
PCA: Principal component analysis
RNAseq: RNA sequencing
RP: Reactome pathway
RT-qPCR: Reverse transcription-quantitative polymerase chain reaction
RTT: Rett syndrome
RTT-like: Rett-like
TF: Transcription factors
ChEA3: WP: WikiPathways
XCI: X-chromosome inactivation
