# Supplementary material for: Identification of molecular signatures and pathways involved in Rett syndrome using a multi-omics approach
Source: Hum Genomics. 2023 Sep 15;17:85. doi: 10.1186/s40246-023-00532-1 (PMC10503149; doi:10.1186/s40246-023-00532-1)

## Slide 1
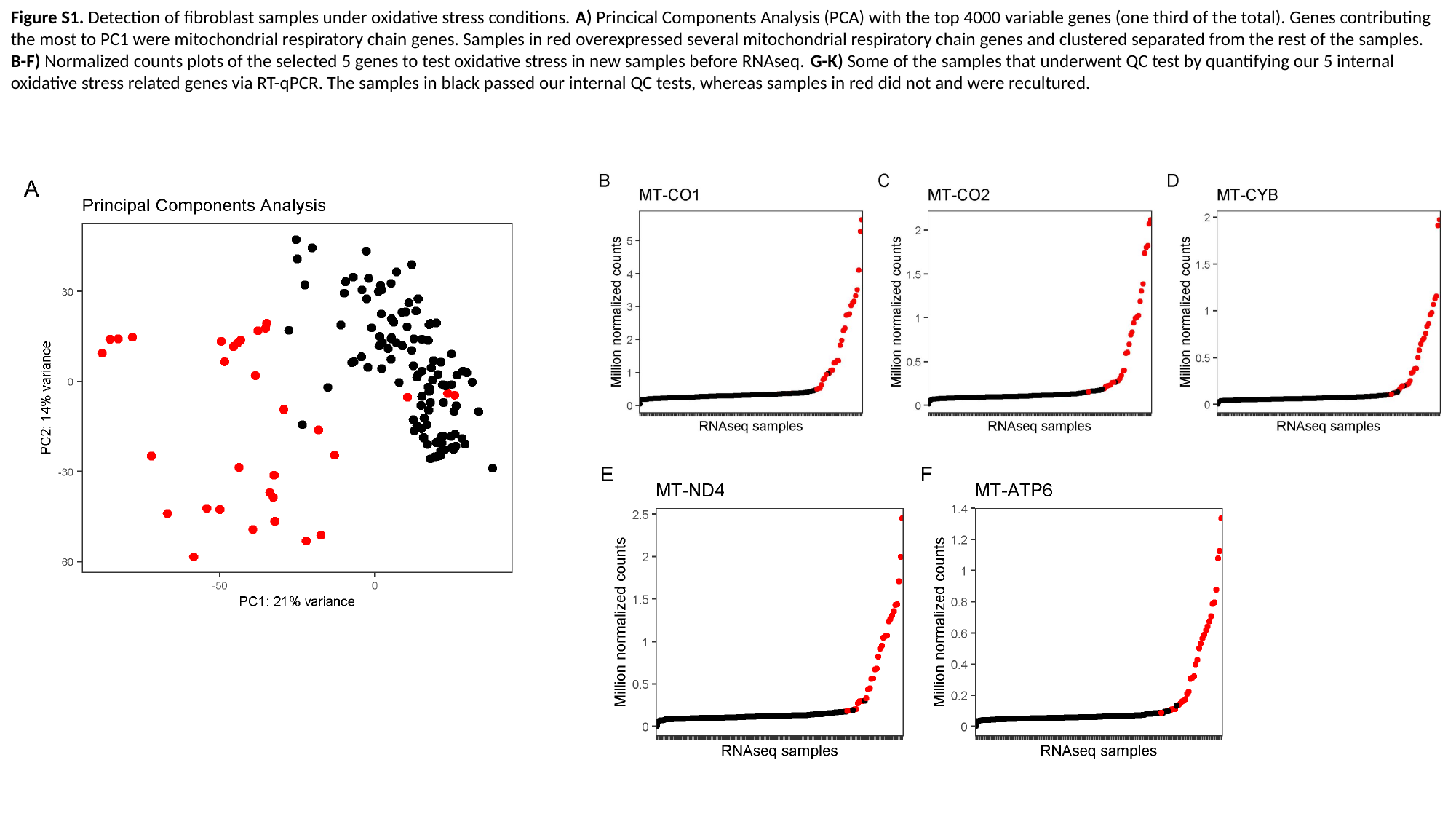

Figure S1. Detection of fibroblast samples under oxidative stress conditions. A) Princical Components Analysis (PCA) with the top 4000 variable genes (one third of the total). Genes contributing the most to PC1 were mitochondrial respiratory chain genes. Samples in red overexpressed several mitochondrial respiratory chain genes and clustered separated from the rest of the samples. B-F) Normalized counts plots of the selected 5 genes to test oxidative stress in new samples before RNAseq. G-K) Some of the samples that underwent QC test by quantifying our 5 internal oxidative stress related genes via RT-qPCR. The samples in black passed our internal QC tests, whereas samples in red did not and were recultured.

## Slide 2
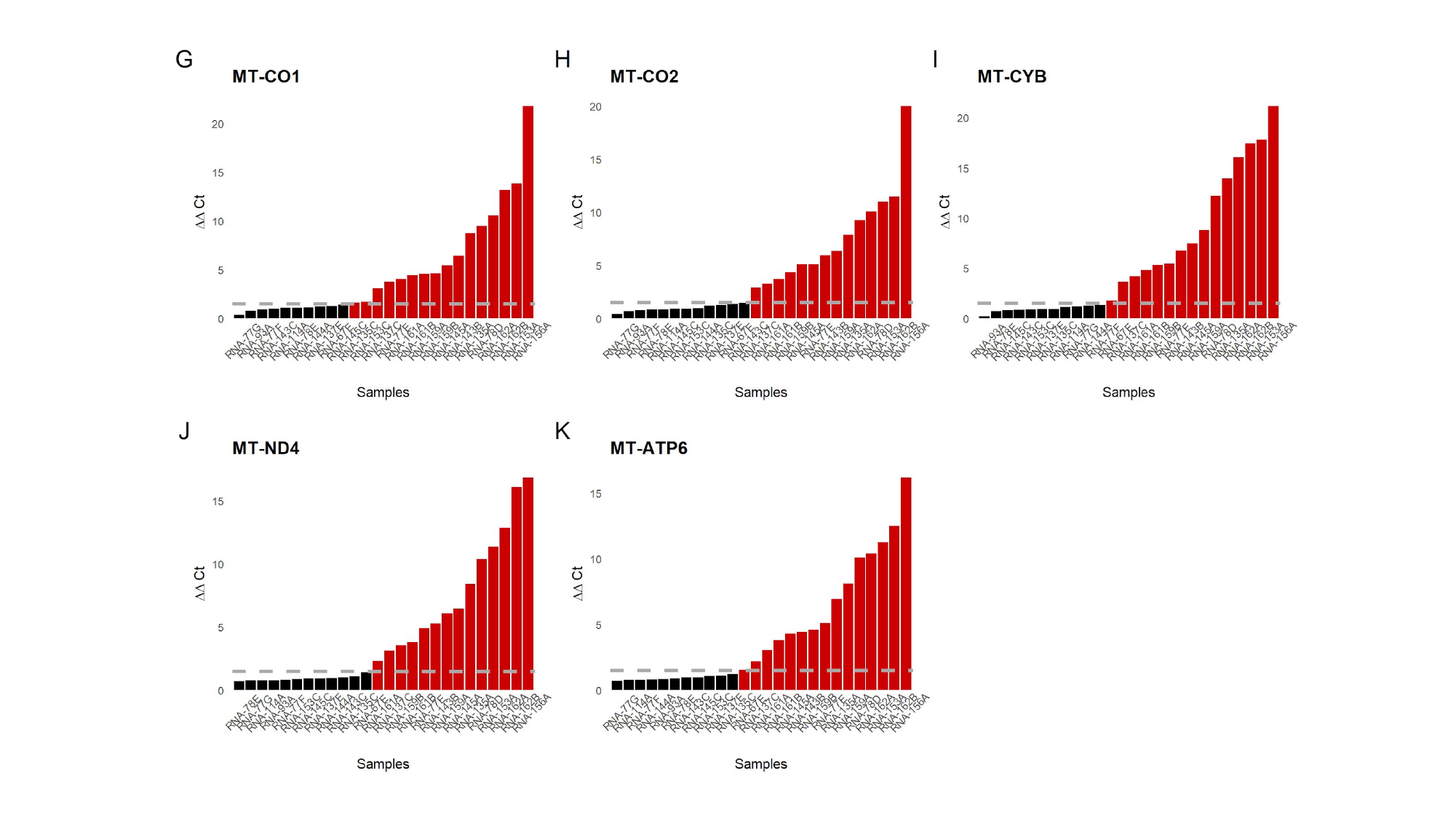

Supplement: Supplementary file 1 — Additional file 1: Fig. S1. Detection of fibroblast samples under oxidative stress conditions. [file 40246_2023_532_MOESM1_ESM.pptx]
