# Supplementary material for: Identification of molecular signatures and pathways involved in Rett syndrome using a multi-omics approach
Source: Hum Genomics. 2023 Sep 15;17:85. doi: 10.1186/s40246-023-00532-1 (PMC10503149; doi:10.1186/s40246-023-00532-1)

## Slide 1
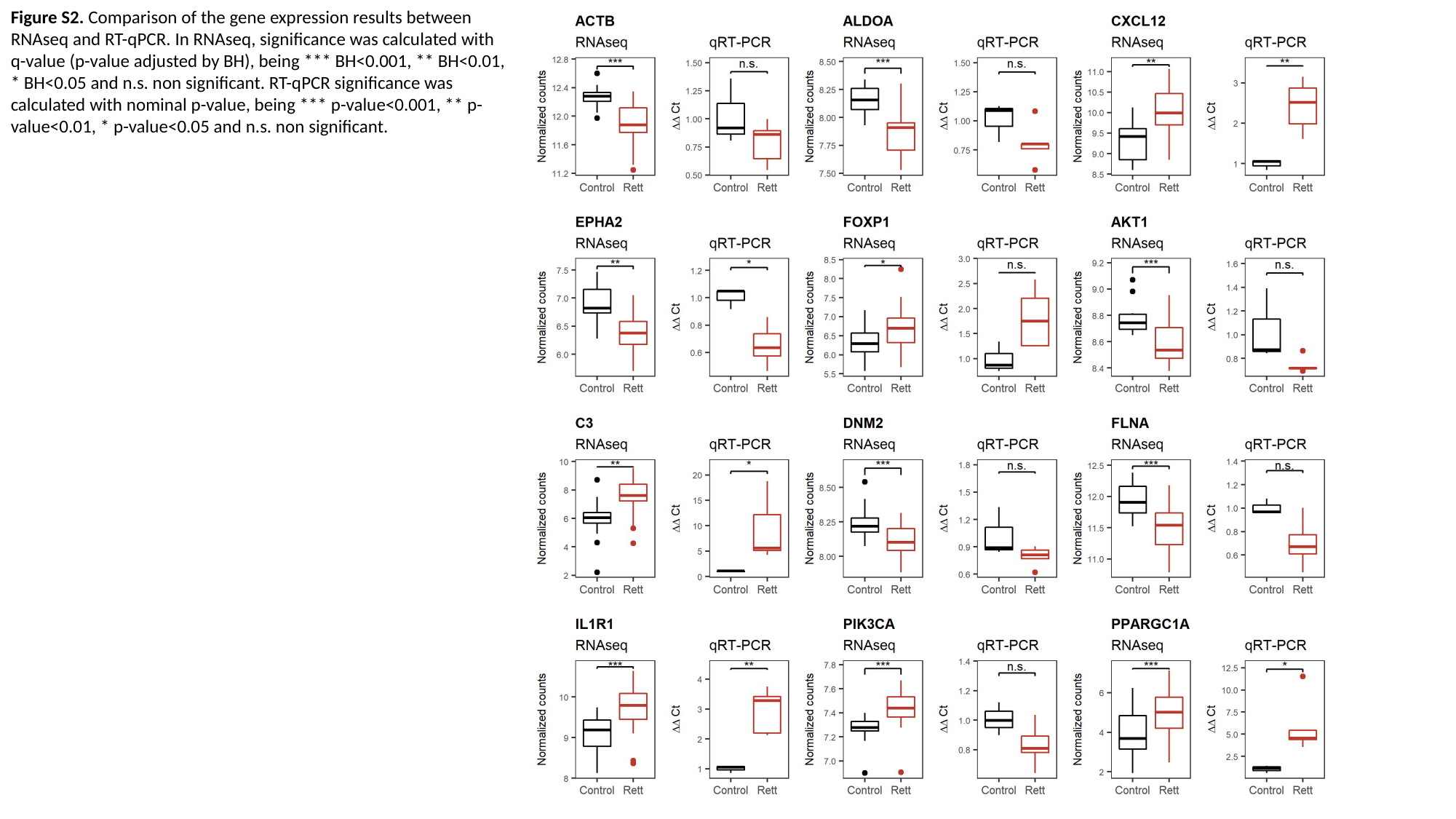

Figure S2. Comparison of the gene expression results between RNAseq and RT-qPCR. In RNAseq, significance was calculated with q-value (p-value adjusted by BH), being *** BH<0.001, ** BH<0.01, * BH<0.05 and n.s. non significant. RT-qPCR significance was calculated with nominal p-value, being *** p-value<0.001, ** p-value<0.01, * p-value<0.05 and n.s. non significant.

## Slide 2
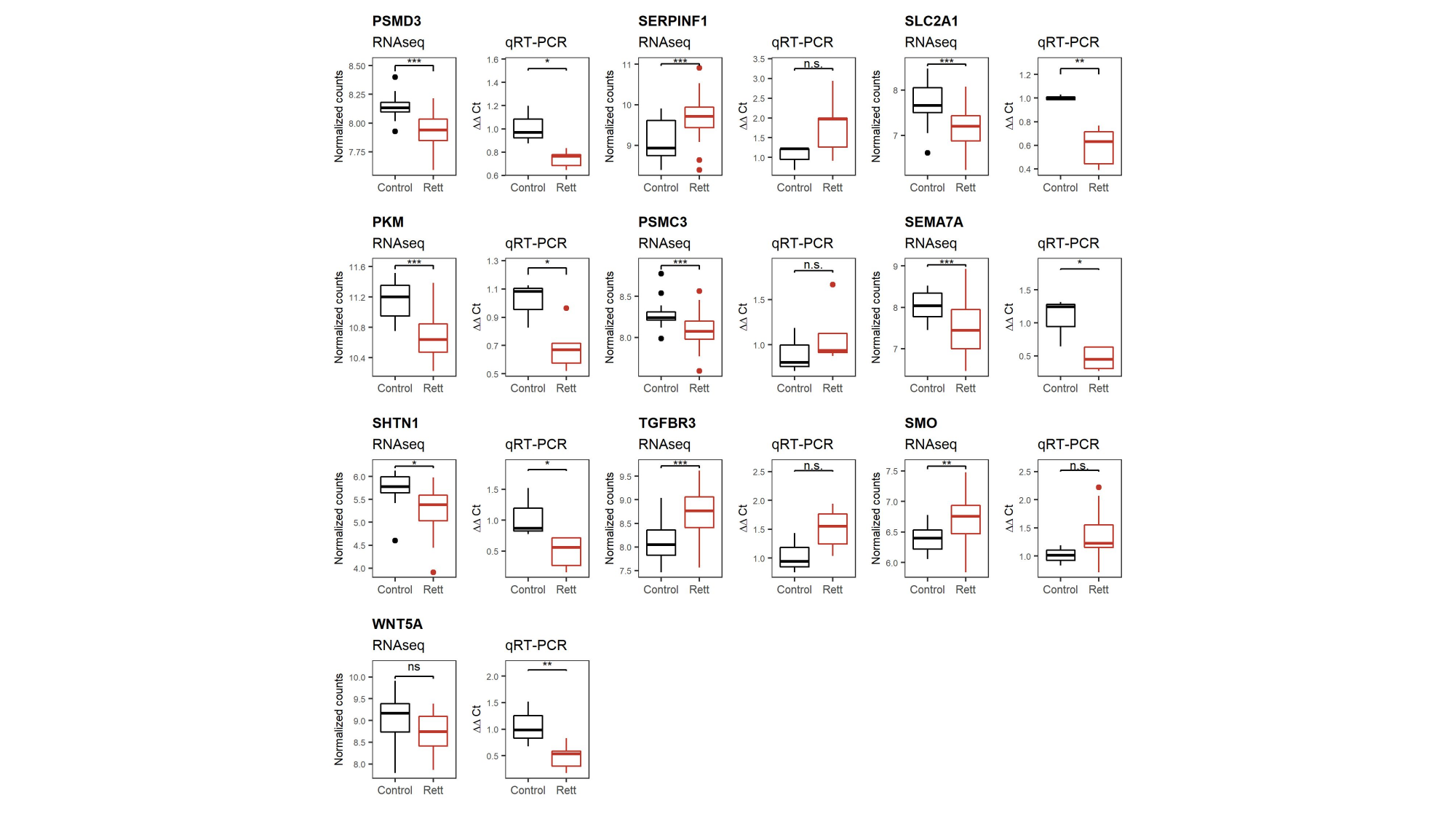

Supplement: Supplementary file 2 — Additional file 2: Fig. S2. Comparison of the gene expression results between RNAseq and RT-qPCR. [file 40246_2023_532_MOESM2_ESM.pptx]
