# Supplementary figures and images for: Identification of molecular signatures and pathways involved in Rett syndrome using a multi-omics approach
Source: Hum Genomics. 2023 Sep 15;17:85. doi: 10.1186/s40246-023-00532-1 (PMC10503149; doi:10.1186/s40246-023-00532-1)

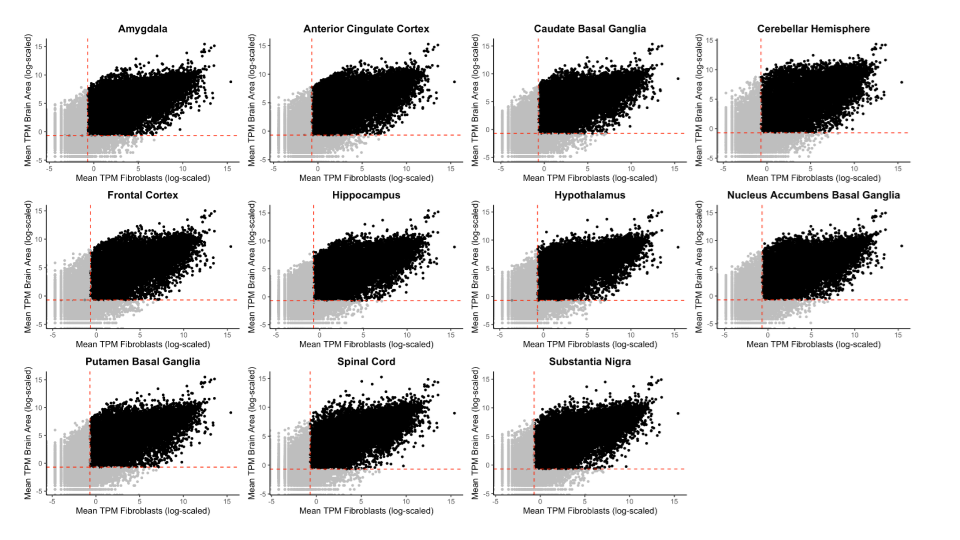

Supplement: Supplementary file 3 — Additional file 3: Fig. S3. Comparison of genes from Genotype-Tissue Expression (GTEx) project and mean TPM (Transcripts per Kilobase Million) in fibroblast cultured cells in RNAseq and RT-qPCR. [file 40246_2023_532_MOESM3_ESM.png]
